# The association between stent design and patient exercise intensity: structural coupling effects and hemodynamic analysis

**DOI:** 10.3389/fbioe.2024.1514929

**Published:** 2024-12-18

**Authors:** Rui Lv, Daochun Li, Shiwei Zhao, Peng Shu, Jinwu Xiang

**Affiliations:** School of Aeronautic Science and Engineering, Beihang University, Beijing, China

**Keywords:** in-stent restenosis, stent design, hemodynamics, connector, exercise intensity

## Abstract

**Introduction:**

In-stent restenosis remains a significant challenge in coronary artery interventions. This study aims to explore the relationship between exercise intensity and stent design, focusing on the coupled response of the stent structure and hemodynamics at different exercise intensities.

**Methods:**

A coupled balloon-stent-plaque-artery model and a fluid domain model reflecting structural deformation were developed to investigate the interaction between coronary stents and stenotic vessels, as well as their impact on hemodynamics. The study examines the influence of stent connectors on the mechanical response of both the plaque and the coronary artery, with hemodynamic analyses conducted under three exercise intensities: rest, moderate exertion, and maximal exertion.

**Results:**

The model effectively simulates the gradual expansion of the stent, plaque, and artery, as well as the recoil behavior post-expansion. The gradual adaptation of the stent to the plaque during the initial expansion phase helps mitigate the adverse effects of the dog-boning phenomenon. Areas of low time-averaged wall shear stress (TAWSS) and high relative residence time (RRT) are observed at both ends and near the stent, with a general decreasing trend as exercise intensity increases. Additionally, the study quantifies the changes in hemodynamic characteristics across different physiological states. Specifically, the areas of low TAWSS and high RRT are significantly reduced during moderate exertion, with no further substantial reduction observed at maximal exertion.

**Discussion:**

These findings provide valuable insights for the design of stent connectors and offer guidance on optimal exercise intensity for patients undergoing stent interventions. Future research, combining dynamic vascular wall deformation and advanced imaging techniques, could lead to more precise and effective stent designs tailored to individual patients.

## 1 Introduction

Coronary stenting is a widely used intervention for treating cardiovascular diseases resulting from arterial stenosis. However, the issue of in-stent restenosis remains a significant challenge that is difficult to fully prevent (Hu and Jiang, 2022; Su et al., 2022; Kapnisis et al., 2023; Giakoumi et al., 2024; Townsend et al., 2022). To address these challenges, considerable attention has been focused on the complex structural interactions and hemodynamic changes induced by coronary stent implantation (Qu et al., 2022; Park et al., 2012; Demanget et al., 2013; Qi et al., 2024; da Silva et al., 2023).

The process of stent expansion can easily lead to damage to plaques and the vessel wall, contributing to in-stent restenosis (Kapnisis et al., 2014; Timmins et al., 2008; Garg and Serruys, 2010; Morlacchi and Migliavacca, 2013; Pant et al., 2012). The structural geometry and size of the stent are key factors influencing the overall stress applied to both the plaque and the vessel (Petrini et al., 2004; Bressloff et al., 2016). Mechanical testing methods, such as radial loading, bending, and torsion, are commonly employed to quantify and assess stent performance (Ormiston et al., 2018). Recoiling and dog-boning have been shown to be closely related to stent length and thickness (Migliavacca et al., 2002). Furthermore, the flexibility of a stent is largely determined by the design of its connectors (Mori et al., 2005). However, evaluating stent performance based solely on mechanical testing has limitations. Research has increasingly focused on creating realistic environments where the stent interacts with balloons, plaques, and arterial walls (Wu et al., 2007; Karimi et al., 2014). Understanding the coupling effects between the stent and the stenotic vessel during expansion is crucial for guiding stent design improvements (Wu et al., 2007). Moreover, stent design must take into account the type of plaque, whether calcified or cellular (Karimi et al., 2014). Multi-objective optimization and topology optimization of stent geometries have proven to significantly enhance the performance of coronary stents (Pant et al., 2011; Kapoor et al., 2023; Xue et al., 2020; Gharleghi et al., 2021).

Research has demonstrated a correlation between hemodynamic alterations induced by stent interventions and the development of neointimal hyperplasia (Kapnisis, et al., 2019). Key stent design parameters, such as ring spacing, angle, and thickness, play a significant role in influencing hemodynamics (He et al., 2005; Hsiao et al., 2012). Reducing the number of stent rings, for instance, has been shown to improve hemodynamic performance (Gundert et al., 2012). Parameters like stent spacing and thickness are closely associated with wall shear stress (WSS) (Beier et al., 2016). Furthermore, the design of the stent connectors has a substantial impact on the distribution of low WSS areas, which are critical to restenosis development (Pant et al., 2012). In addition, compared to traditional cylindrical designs, tapered stents have been found to offer superior potential in preventing restenosis (Yu et al., 2016). While these studies provide valuable insights, they often overlook the deformation of the vessel wall caused by stent expansion under real-world conditions. Incorporating the effects of stent-induced vessel deformation into hemodynamic analyses could offer more accurate and clinically relevant guidance (Morris et al., 2018). Research has also confirmed that stent placement and expansion significantly influence WSS distribution (Morlacchi et al., 2011). Moreover, the shape and curvature of coronary arteries are closely linked to hemodynamic parameters, further emphasizing the complexity of the issue (Martin et al., 2014; Wei et al., 2019; Shen et al., 2021).

The connector plays a crucial role in ensuring the flexibility of the stent, making it an essential component of the stent structure. However, there remains limited research regarding the influence of connectors on the structural coupling response and the resulting hemodynamic characteristics after stent deformation. Additionally, most existing studies primarily consider blood flow velocity under specific physiological conditions as the input parameter, without fully addressing how variations in different physiological states affect hemodynamic behavior. In this study, we develop a comprehensive coupling model that incorporates the balloon, stent, plaque, and artery. The interactions of stents featuring S-shaped, I-shaped, C-shaped, and W-shaped connectors (designated as S-stent, I-stent, C-stent, and W-stent) with the plaque and artery are examined. In addition, the hemodynamic effects of different stents are compared based on the deformed stent-plaque-artery model, employing three real physiological states (including varying heart rates) as boundary conditions. This study aims to provide new insights into the coupling effects of stent connectors on arterial hemodynamics under realistic physiological scenarios.

## 2 Evaluation index

The performance of the stent is assessed by considering the interaction effects between the stent, plaque, and arterial wall, as well as two key mechanical phenomena: stent dog-boning (*F*_d_) and recoiling (*F*_r_). Dog-boning refers to the tendency of the stent to expand more at its ends than in the central region during deployment, which can lead to vessel damage and should be minimized. Recoiling describes the phenomenon in which the stent reverts to its original shape after unloading, potentially compromising its ability to provide sustained support to the artery. The values of *F*_d_ and *F*_r_ can be determined from Equations 1 and 2, respectively (Wei et al., 2019).

$$F_{d}=\frac{L_{e}-L_{m}}{L_{m}}$$ (1)

$$F_{r}=\frac{L_{\max}-L_{m}}{L_{m}}$$ (2)

where *L*_e_ and *L*_m_ represent the average diameters of the two ends and the middle of the stent after final stabilisation, respectively. *L*_max_ represents the average diameter of the middle section corresponding to the maximum expansion of the stent.

Wall shear stress (WSS) refers to the instantaneous viscous stress exerted by blood flow on the walls of both the stent and the vessel. Numerous studies have highlighted the association between regions of low WSS (typically less than 0.5 Pa) and an increased risk of vascular complications, such as intimal hyperplasia, which may subsequently lead to restenosis following stent implantation (Qi et al., 2024; Wei et al., 2019). However, WSS exhibits significant temporal fluctuations throughout the cardiac cycle. Additionally, increased exercise intensity shortens the cardiac cycle (Ellwein et al., 2017), resulting in variations in instantaneous WSS. These fluctuations complicate the comparison and interpretation of WSS. Time-averaged wall shear stress (TAWSS) overcomes this issue by averaging WSS over the entire cardiac cycle, which helps to mitigate the effects of variations in cardiac cycle duration. By smoothing out the effects of these variations, TAWSS provides a more stable and consistent measure of shear stress that is less affected by fluctuations in heart rate or other transient factors. Therefore, TAWSS is a more appropriate index for evaluating the long-term hemodynamic environment. WSS and TAWSS can be obtained from Equations 3, 4, respectively (Beier et al., 2016; Pant et al., 2010).

$$\text{WSS}=n_{i}\times \tau_{\text{ij}}$$ (3)

$$\text{TAWSS}=\frac{1}{t}\int_{0}^{t}\left|\text{WSS}\right|dx$$ (4)

where n_i_ represents the normal vector of the wall formed by the stent and the vessel surface and $\tau_{\text{ij}}$ represents the viscous stress tensor. *t* represents the total time during the calculation cycle.

Besides TAWSS, the oscillatory shear index (OSI) and relative residence time (RRT) are critical hemodynamic parameters, commonly used to assess disturbed blood flow and predict the development of local atherosclerotic plaques. OSI values greater than 0.1 are associated with an increased risk of stenosis, whereas RRT values exceeding 8 Pa^−1^ are considered a critical threshold for predicting in-stent restenosis (Wei et al., 2019; Lagache et al., 2021). OSI and RRT are determined by Equations 5 and 6, respectively.

$$\text{OSI}=\frac{1}{2}\left(1-\frac{\left|\int_{0}^{t}\text{WSSd}x\right|}{\int_{0}^{t}\left|\text{WSS}\right|dx}\right)$$ (5)

$$\text{RRT}=\frac{1}{\left(1-2\times \text{OSI}\right)\times \text{TAWSS}}$$ (6)

## 3 Numerical modeling

### 3.1 Geometric model

**FIGURE 1:**
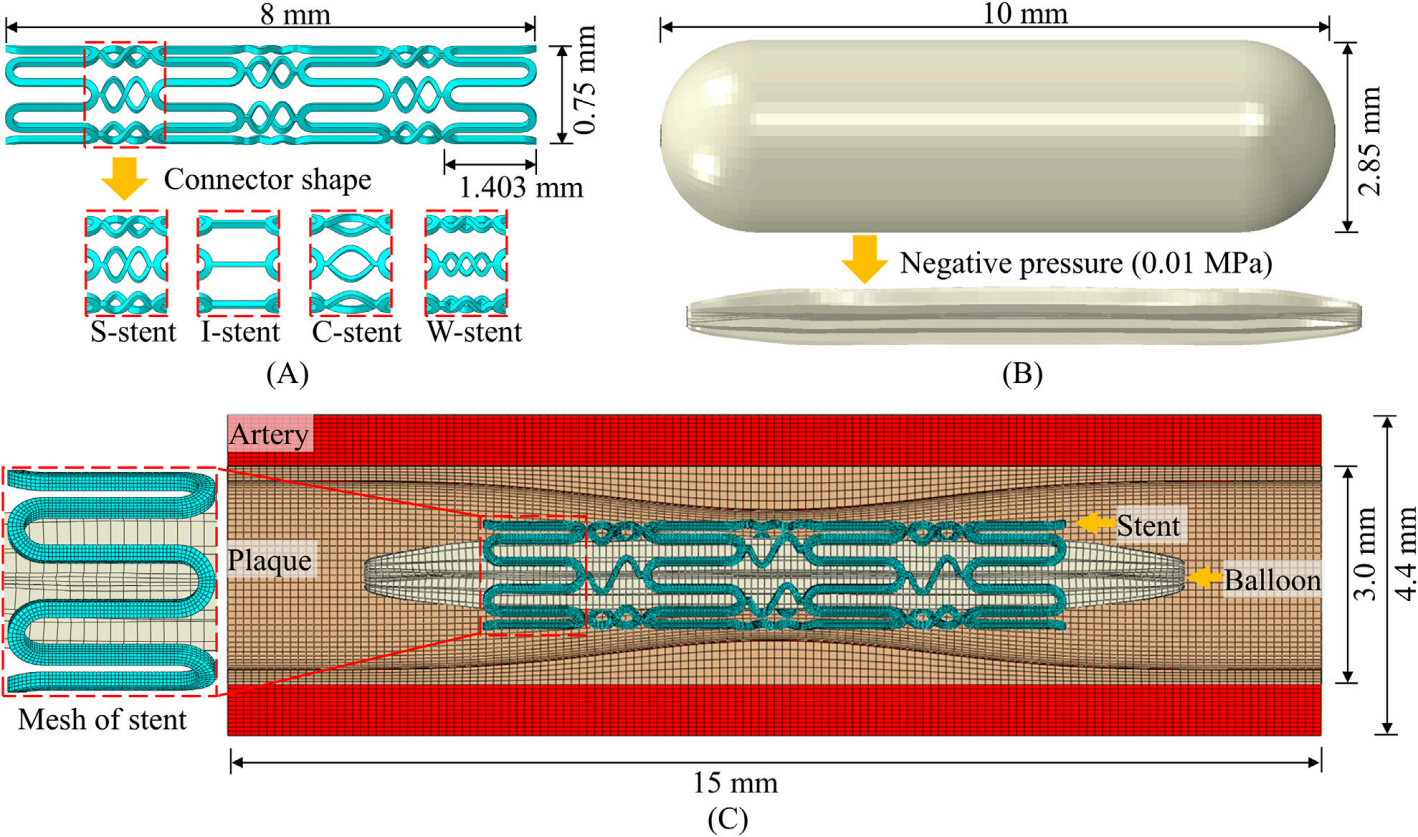
Numerical model. **(A)** Stent. **(B)** Balloon. **(C)** Finite element model of assembly.

The geometry of the stent, plaque as well as the artery is designed using SolidWorks. The meshing of the parts and the running of the simulation are based on ABAQUS/Explicit. The structural design and geometry of the vascular stent are based on the CYPHER stent. As shown in Figure 1A, the overall length of the stent, the circumferential ring length, and the diameter in the crimped state are 8 mm, 1.403 mm, and 0.75 mm, respectively. The influence of connector design on the mechanical properties of stents has been extensively studied (Pant et al., 2010; Pant et al., 2012), and this study specifically investigates the effect of connector shape on stent performance. The shapes of connectors included four types: S-shaped, I-shaped, C-shaped and W-shaped, and the corresponding vascular stents are named S-stent, I-stent, C-stent and W-stent, respectively. It can be seen from Figure 1B that the balloon has a nominal diameter of 2.85 mm and a nominal length of 10 mm. The structural geometry of the assembly consists of the balloon, stent, plaque, and artery, as shown in Figure 1C. The outer diameter, thickness, and total length of the artery are 4.4 mm, 0.7 mm, and 15 mm, respectively. The curves corresponding to the plaque use the modified function (Pant et al., 2012), as shown in Equation 7

$$y=h+\left(0.6-0.2\right){\left[\sin\pi {\left(\frac{x}{L}\right)}^{\left(-\ln2/\ln0.5\right)}\right]}^{5}\left(0<x<15\text{ mm}\right)$$ (7)

where *h* represents the base thickness of the plaque with a size of 0.2 mm and *L* represents the total length of the plaque with a size of 15 mm.

The stent, artery and plaque are discretized using 8-node linear brick elements with reduced integration, and the balloon is discretized by 4-node quadrilateral membrane elements with reduced integration. Following a mesh convergence analysis, four layers of elements are assigned along the thickness direction of the stent, resulting in approximately 53,000 elements for the stent. The total number of elements for the balloon, plaque, and artery are 8,500, 56,000, and 134,400, respectively. The radial and tangential displacements at the ends of the balloon are constrained to simulate the connection to the catheter, and other boundary conditions are similar to those used by Wei et al. (2019). A negative pressure of 0.01 MPa is applied to the inner surface of the balloon to narrow it into the stent to simulate the stent being compression gripped onto the balloon (Pant et al., 2012; Wei et al., 2019). This is followed by the application of a positive internal pressure of 1.2 MPa to induce stent expansion. The expansion process is divided into three distinct phases: loading, holding, and unloading (Pant et al., 2012).

The surface-to-surface contact algorithm is employed for the self-contact of the balloon and the mutual contact (Balloon-stent, stent-plaque) between different components, and the friction coefficient is 0.2. Tie constraint is set on the contact surface of the plaque with the artery to ensure consistent deformation (Wei et al., 2019). Fixed constraints are set at both ends of the artery and plaque.

### 3.2 Material model

The stent material utilized medical 316L stainless steel, which has been applied on both Palmaz Schatz and Cypher stents (Pant et al., 2012). The elastic-plastic constitutive model is adopted to model the stent. The Young’s modulus of the stent material is 196 GPa, the Poisson’s ratio is 0.3, and the yield stress is 375 MPa. The stress-strain relationship is derived from the experiment carried out by Murphy et al. (2003). The linear elastic constitutive is employed to define the material properties of the balloon (De Beule et al., 2008). The elastic modulus of the balloon material is 920 MPa, and the Poisson’s ratio is 0.4 (De Beule et al., 2008; Pant et al., 2012). Additionally, the material densities for the stent (7,800 kg/m^3^) and balloon (1,100 kg/m^3^) are taken from the reference (De Beule et al., 2008).

Schiavone et al. (2014) found that the Mooney-Rivlin and Ogden models have similar expansion effects and the material parameters of the Ogden model are more complete. Therefore, the mechanical properties of both plaque and artery are defined using the Ogden hyper-elastic constitutive model, as shown in Equation 8.

$$W=\sum_{j=1}^{N}\frac{2\mu_{j}}{{\alpha_{j}}^{2}}\left({\beta_{1}}^{\alpha_{j}}+{\beta_{2}}^{\alpha_{j}}+{\beta_{3}}^{\alpha_{j}}-3\right)+\sum_{j=1}^{N}\frac{1}{D_{j}}{\left(K-1\right)}^{2j}$$ (8)

where *N* is related to the amount of terms in the model. $\mu_{j},\alpha_{j},D_{j}$ represent the relevant material parameters and their values are shown in Table 1 (Schiavone et al., 2014; Zahedmanesh et al., 2009). The material parameters of the media layer are used for arterial modelling (Pant et al., 2012). $\beta_{j}$ and *K* represent the principal stretches and elastic volume ratio, respectively.

**TABLE 1:** Modeling parameters for the artery and plaque (Schiavone et al., 2014; Zahedmanesh et al., 2009).

Material	$\mu_{1}$	$\mu_{2}$	$\mu_{3}$	$\alpha_{1}$	$\alpha_{2}$	$\alpha_{3}$	$D_{1}$	ρ (kg/mm^3^)
Artery	−1.23	0.88	0.45	16.59	16.65	16.5	5.31e-6	1.066e-6
Plaque	0.093	—	—	8.17	—	—	4.30e-6	1.45e-6

### 3.3 Modeling of computational fluid dynamics

**FIGURE 2:**
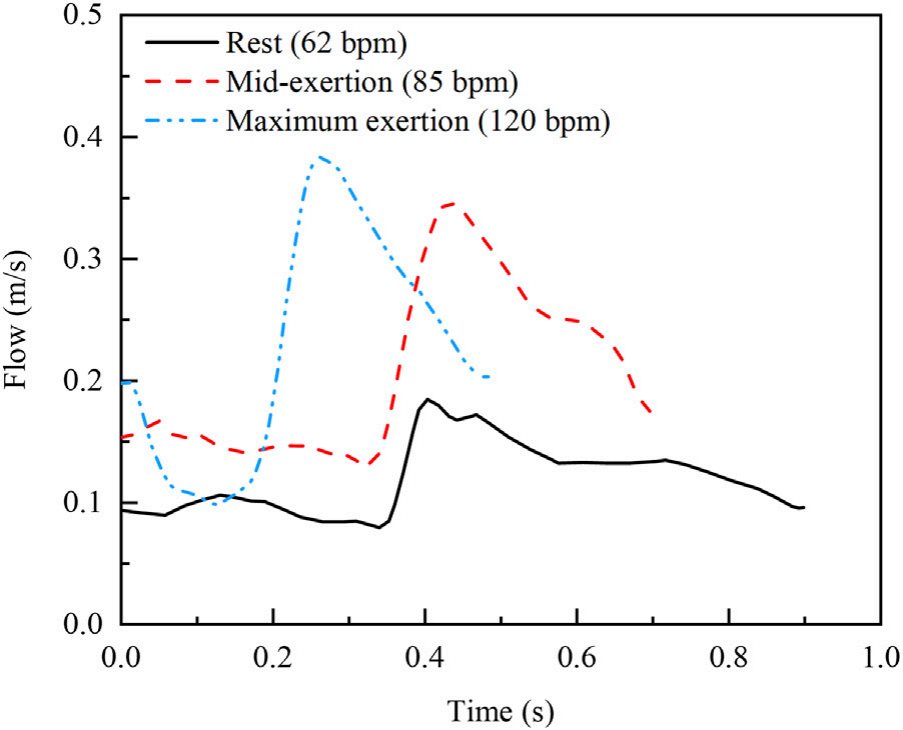
Inlet velocity profiles at three real exercise intensities (Broyd et al., 2015).

Node information is extracted from the deformed stent and plaque structures for the reconstruction of the geometric model. Boolean operations, performed in SolidWorks, are then used to delineate the fluid domains. The meshing of the fluid domains is performed using ANSYS ICEM, and simulations are conducted with ANSYS Fluent. The cavity formed by the stent and plaque is treated as a rigid no-slip boundary, and blood is modelled as an incompressible, laminar, Newtonian fluid. The density of blood is 1,060 kg/m³, and its coefficient of viscosity is 0.0035 Pa·s (Wei et al., 2019). The fluid domain is discretized using Tetra/Mixed elements, and after conducting a mesh sensitivity analysis, the final mesh consists of approximately 3.5 million elements. For the boundary conditions, the outlet pressure is set to zero relative pressure and the blood flow velocity profile is used for the inlet. The time step is set to 0.005 s, with a convergence of 10^–5^. The blood flow velocity profiles corresponding to rest (62 bpm), mid-exertion (85 bpm) and maximum exertion (120 bpm) are shown in Figure 2 (Broyd et al., 2015).

## 4 Results and analysis

The connector is an important part of the stent, and its role is to provide sufficient flexibility for the expanded vascular stent. Based on the developed model, the effects of the connector shape on the structural coupling response and hemodynamic characteristics are studied.

### 4.1 Analysis of structural coupling effects

#### 4.1.1 Analysis of the stent expansion process

**FIGURE 3:**
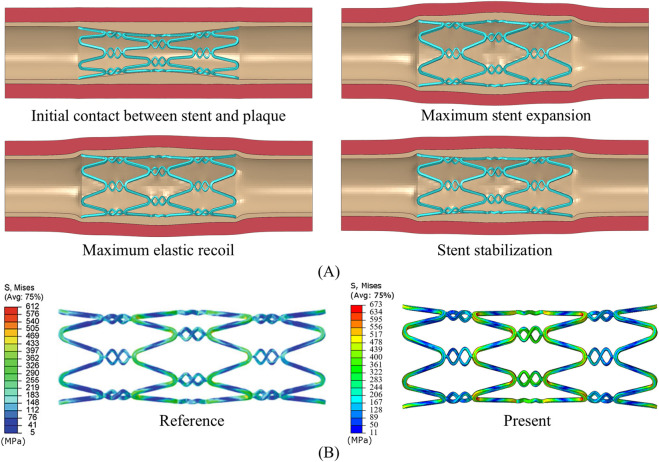
**(A)** Stent expansion process. **(B)** Final stress distribution of the stent, compared with Pant et al. (2012).

As shown in Figure 3A, the developed model successfully simulates the phenomenon of dog-boning during stent expansion. Initially, as the balloon gradually inflates, both the stent, blood vessels, and plaques reach their maximum expansion. When the balloon begins to recoil, the elastic recoil of the stent, vessel, and plaque becomes evident, peaking as the balloon further deflates. Ultimately, the stent undergoes plastic deformation due to the combined forces of the plaque and the arterial wall, reaching a stable configuration. This expansion process closely resembles the findings presented by Pant et al. (2012). In addition, the stress distribution after the stabilization of the stent is compared with the reference, as shown in Figure 3B. It can be observed that there are obvious stress concentrations at both the connector and the ring. The stress concentration in the middle of the stent is more pronounced due to the compression effect of the central plaque. The maximum stress in the stent differs by 9.97% from the reference. In summary, the model developed can be used to study the coupling of the stent to the stenotic vessel.

#### 4.1.2 Mechanical response of stents

**FIGURE 4:**
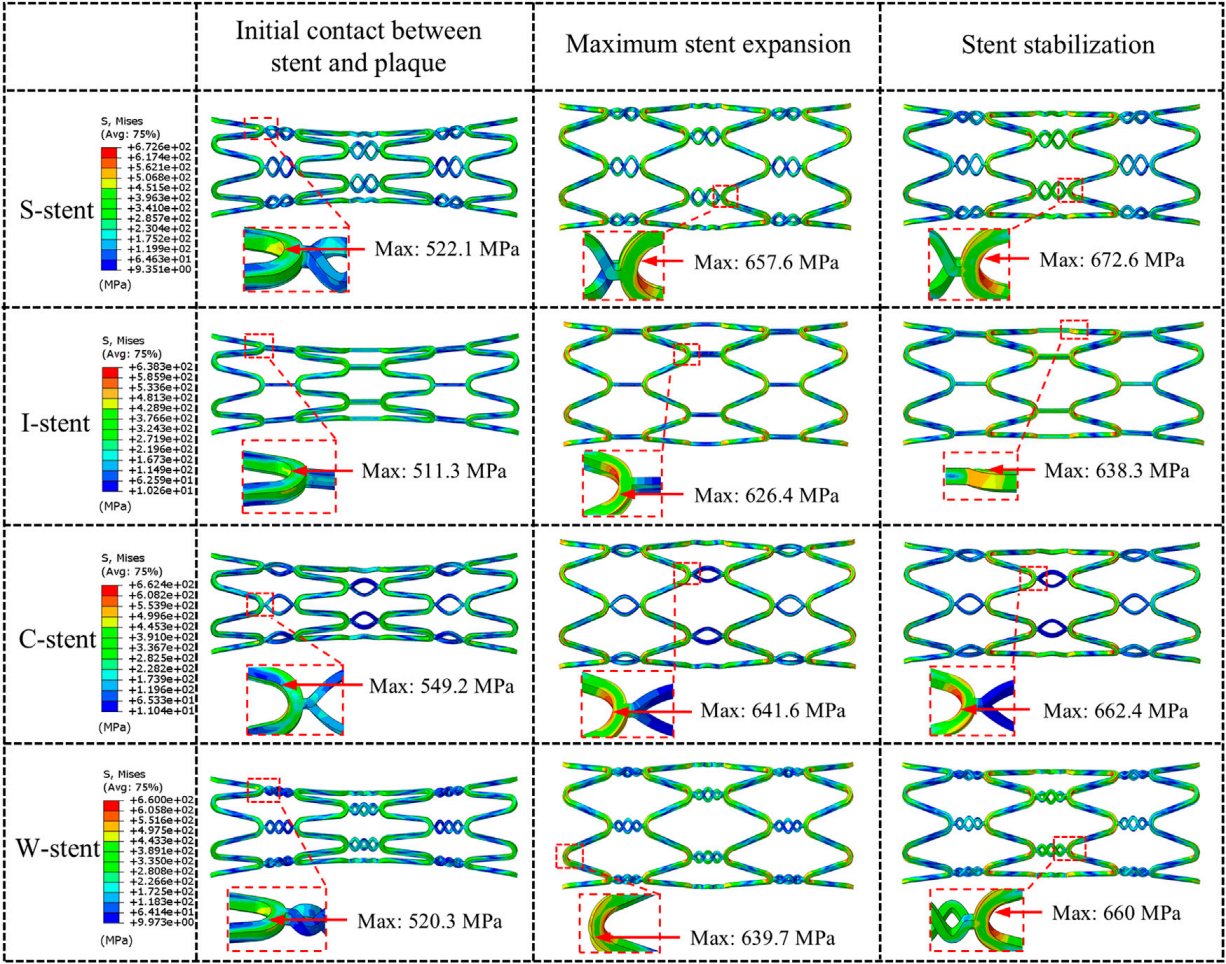
Comparison of the stent expansion process across different designs.

Three stages of mechanical response are selected for comparison: initial contact between stent and plaque, maximum stent expansion, and the stent stabilization phase. As shown in Figure 4, all stents exhibit a significant dog-boning effect at initial contact with the plaque, with the maximum stress occurring at the junctions between the rings and connectors at both ends of the stent. This is due to the balloon expanding more rapidly at the ends compared to the center during the initial stage of expansion. Over time, the dog-boning effect is gradually alleviated through the combined action of the balloon, plaque, and artery. The peak stress values shift from the ends to the center of the stent, reflecting the higher degree of stenosis in the middle of the artery, which leads to a more concentrated region of high stress at the center of the stent. Notably, throughout the entire expansion process, the I-stent exhibits the lowest peak stress values compared to the other three stent types.

**FIGURE 5:**
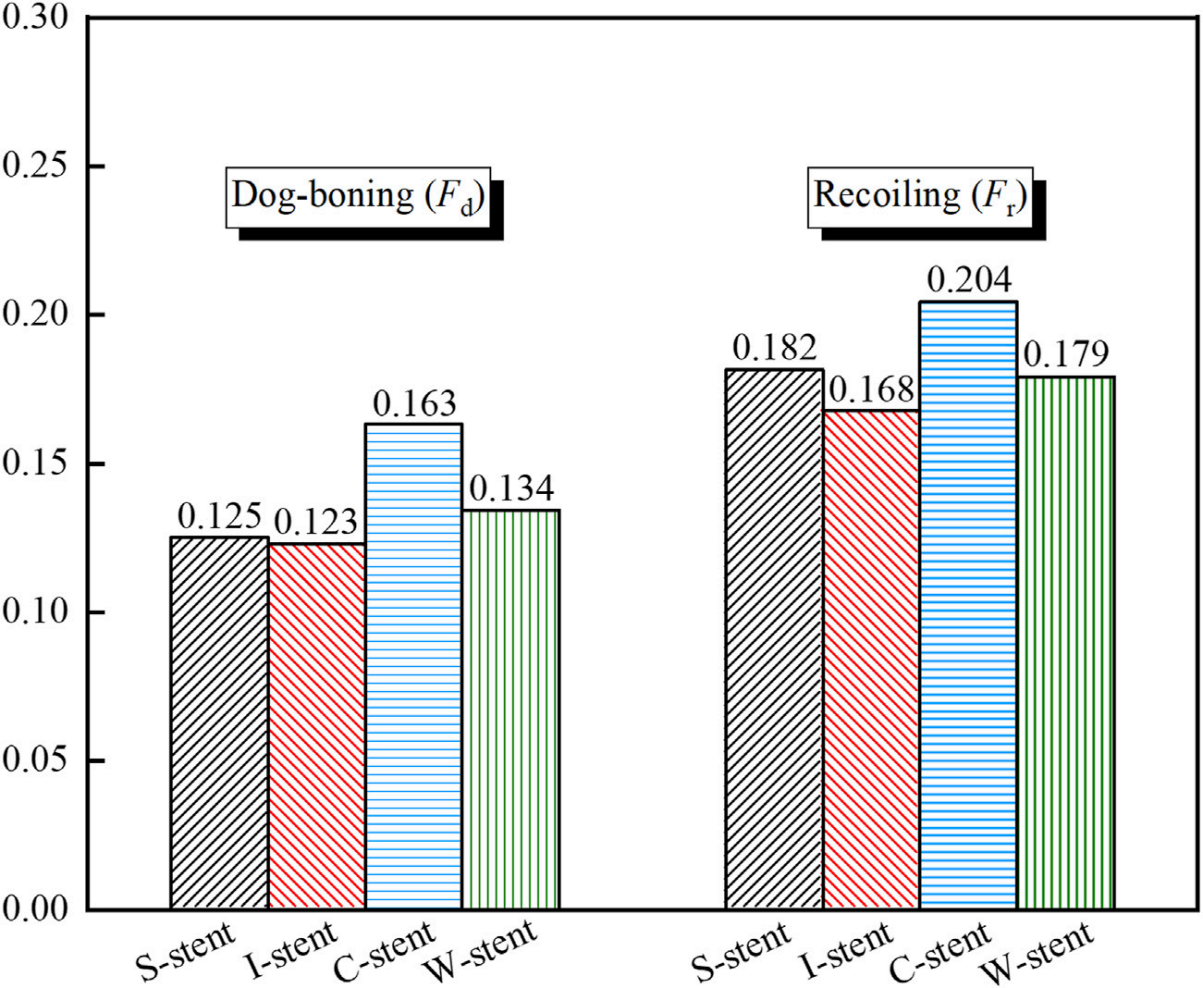
Dog-boning (*F*_d_) and recoiling (*F*_r_) characteristics for different stent designs.

As shown in Figure 5, the dog-boning and recoiling effects are used to evaluate the performance of the stent. The dog-boning values for the S-stent (0.125) and I-stent (0.123) are relatively close, with the I-stent exhibiting the minimum dog-boning effect and the C-stent (0.163) showing the maximum. Additionally, the recoiling values for the I-stent (0.168) and C-stent (0.204) are the minimum and maximum, respectively. The recoiling values for the S-stent (0.182) and W-stent (0.179) are also similar. Overall, the S-stent, I-stent, and W-stent perform better in terms of both dog-boning effect and recoiling compared to the C-stent.

#### 4.1.3 Mechanical response of plaques and arteries

**FIGURE 6:**
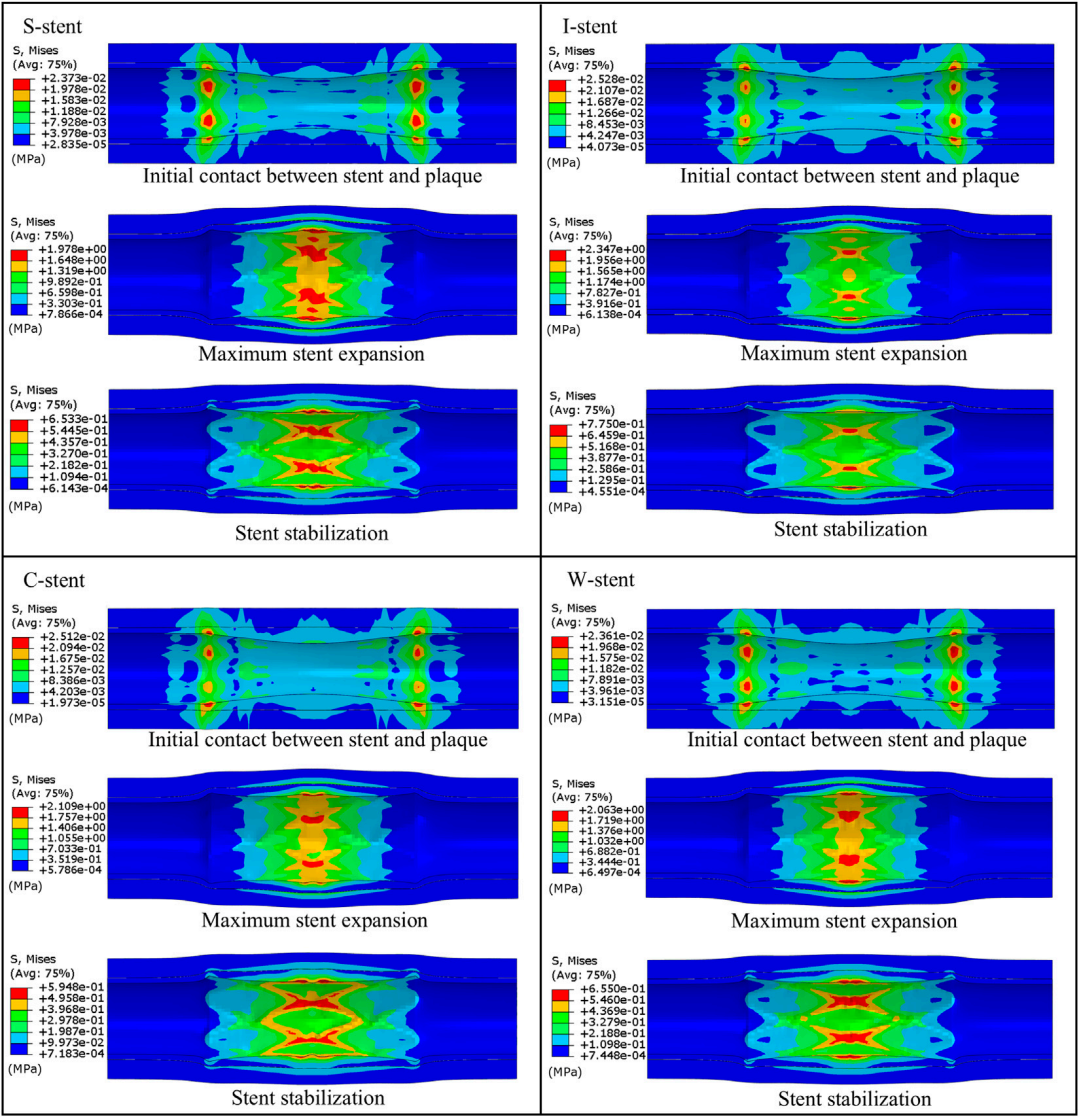
Comparison of the expansion process of plaques and arteries under different stent designs.

The stress clouds of plaques and arteries at different stages of expansion are shown in Figure 6. It is evident that upon contact with the plaque, all four types of stents induce noticeable stress concentration at both ends of the plaque. The maximum stress observed with the I-stent is the highest, followed by the C-stent, S-stent, and W-stent, in that order. As the stent continues to expand, both the maximum stress experienced by the plaque and the artery and the area of stress concentration increase significantly. Furthermore, the region of stress concentration progressively shifts from the ends toward the center of the plaque. When the stent reaches its maximum expansion, the I-stent generates the highest stress in both the plaque and the artery. Specifically, the stresses induced by the S-stent, C-stent, and W-stent are 15.7%, 10.1%, and 12.1% lower, respectively, compared to those caused by the I-stent. This suggests that the I-stent is more likely to induce damage to the vessel wall due to higher localized stresses.

Additionally, a marked reduction in stress is observed when the stent is retracted to a stable position. During the expansion process, stress values in both the plaque and artery increase significantly when the stent reaches its maximum diameter, with the peak stress localized at the central region of the plaque. This is attributed to the higher degree of stenosis at the center of the plaque, resulting in excessive compression from both the plaque and the stent. Overall, compared to the I-stent and C-stent, the S-stent and W-stent provide a more uniform distribution of stress across the plaque and artery, thereby reducing the likelihood of significant damage to the vessel wall. These findings suggest that S-stents and W-stents may offer advantages in terms of minimizing localized stress and preventing vessel wall injury during stent deployment.

### 4.2 Analysis of hemodynamic characteristics

#### 4.2.1 Effects of connectors on hemodynamics

**FIGURE 7:**
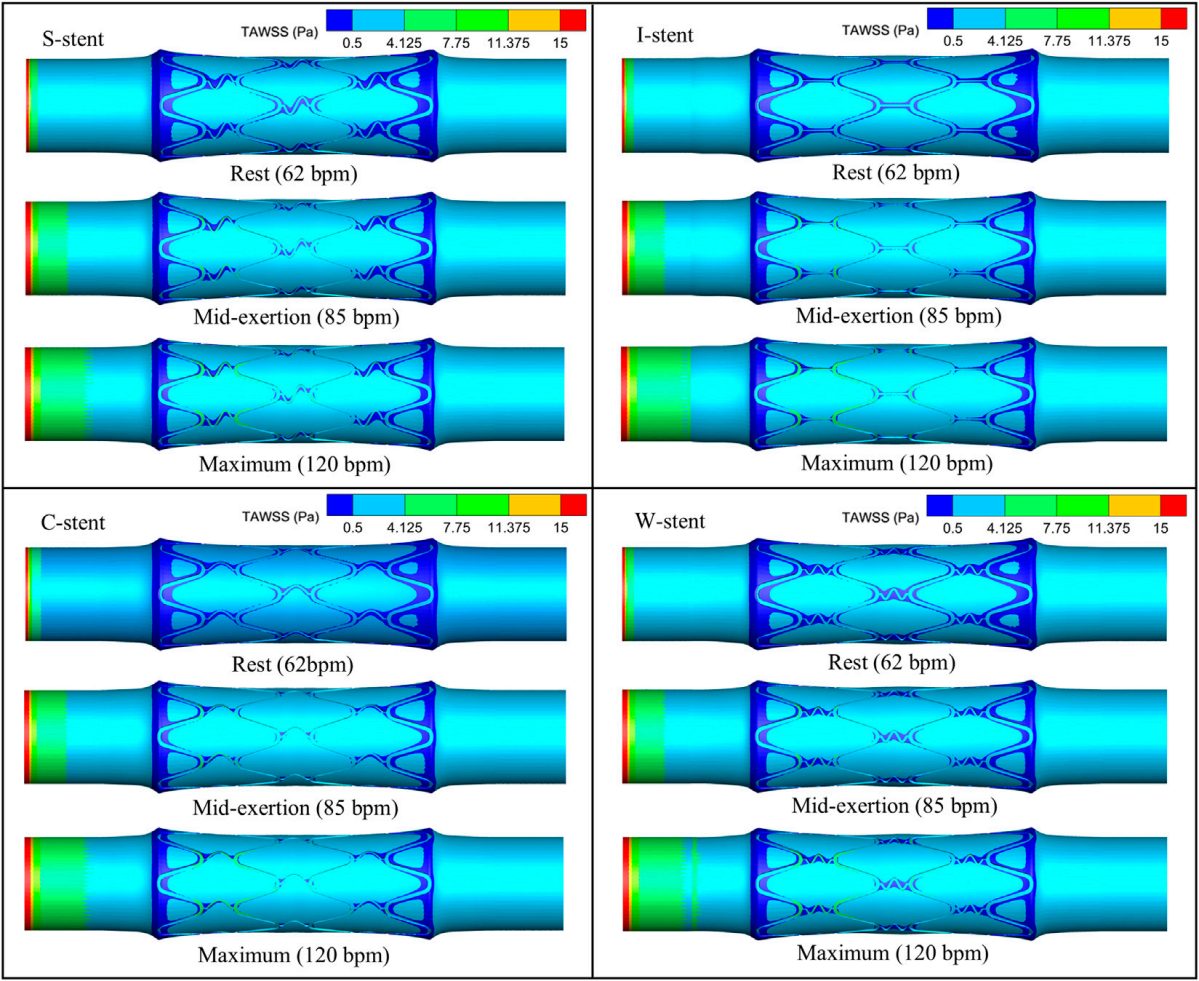
TAWSS distribution corresponding to all stents under different physiological states.

The distribution of TAWSS corresponding to all stents on the vessel surface under different physiological states is shown in Figure 7. It can be observed that the low TAWSS areas are mainly observed at the ends and around the stent. The area of low TAWSS near the connectors of the S-stent and W-stent is larger than that of the I-stent and C-stent. This is mainly due to the different shapes of the connectors, which differentially influence the obstruction of blood flow. With increasing heart rate (from rest to maximum exertion), the low TAWSS area overall shows a decreasing trend, while the high TAWSS (greater than 0.5 Pa) area at the stent position increases significantly.

**FIGURE 8:**
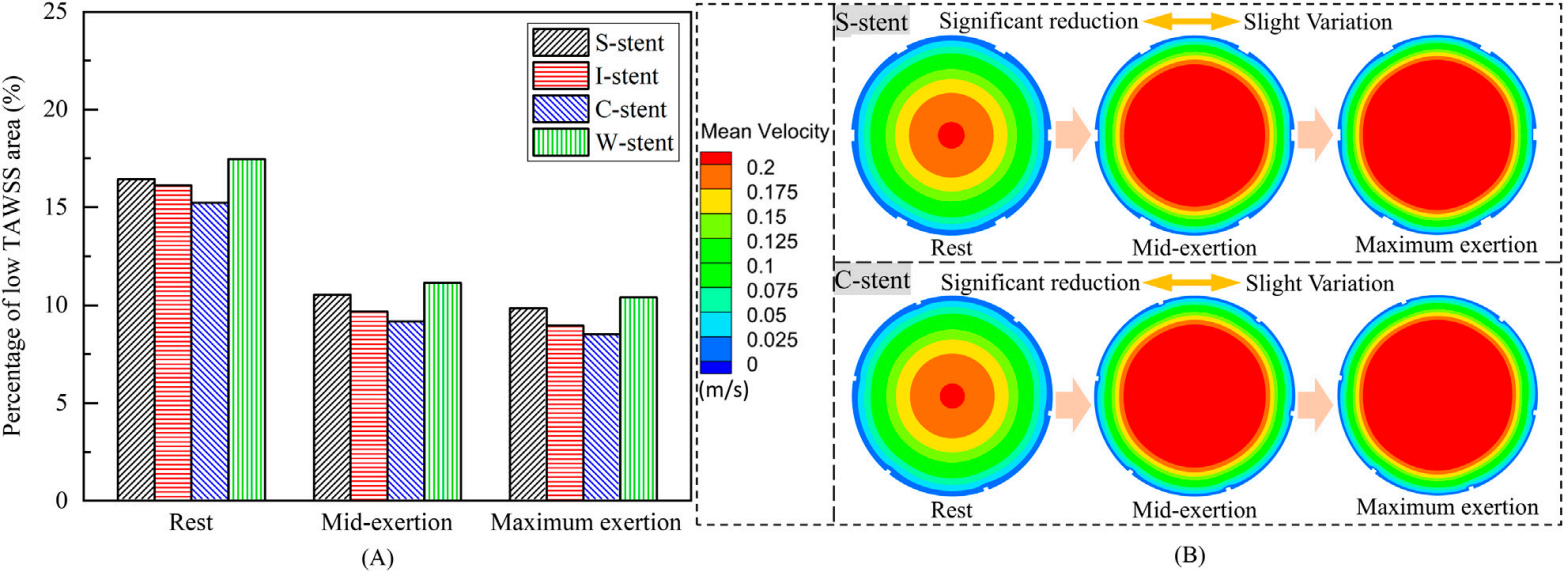
**(A)** Percentage of low TAWSS areas corresponding to different stents. **(B)** Mean velocity in the arterial mid-section.

As shown in Figure 8A, the percentage of the low TAWSS area (less than 0.5 Pa) relative to the total surface area of the fluid domain is used to quantify the differences between stents with different connectors. Compared to the other stents, the C-stent exhibits the smallest percentage of low TAWSS area across all physiological states, followed by the I-stent, S-stent, and W-stent. At rest, the S-stent, I-stent, and W-stent show increases of 7.81%, 6.04%, and 14.63%, respectively, in the low TAWSS area compared to the C-stent. As the heart rate increases (from rest to maximum exertion), the differences between the S-stent and W-stent relative to the C-stent increase, while the difference between the I-stent and C-stent decreases. Overall, the C-stent demonstrates superior hemodynamic performance compared to the other three stent designs.

The influence of physiological changes on the percentage of low TAWSS area is also evident in Figure 8A. The low TAWSS area induced by the S-stent decreases by 35.9% and 40.0% at mid-exertion and maximum exertion, respectively, compared to the resting state. For the I-stent, the low TAWSS area decreases by 40.0% and 44.5% at mid-exertion and maximum exertion, respectively, when compared to rest. These results clearly indicate that transitioning from rest to mid-exertion leads to a significant reduction in the low TAWSS area for both the S-stent and I-stent. However, the reduction in low TAWSS area from mid-exertion to maximum exertion is notably smaller. Furthermore, the changes in low TAWSS area induced by the C-stent and W-stent from rest to maximum exertion follow similar trends to those observed with the S-stent and I-stent.

To explain the underlying mechanisms driving the variation in low TAWSS area across different physiological states, the mean velocity distributions in the middle cross-sections of the vessels for the S-stent and C-stent are analyzed, as shown in Figure 8B. At rest, the highest mean velocity is observed in the center of the cross-section, while a large region of low mean velocity is present along the wall surface. As the physiological state transitions to mid-exertion, the low mean velocity region along the wall surface decreases, and a significant increase in high mean velocity is observed in the central region of the cross-section. This explains the marked reduction in the low TAWSS area during mid-exertion. Additionally, when the physiological state shifts from mid-exertion to maximum exertion, the changes in mean velocity, both in the center and along the wall surface, are relatively small, which accounts for the lack of significant reduction in the low TAWSS area during maximum exertion.

**FIGURE 9:**
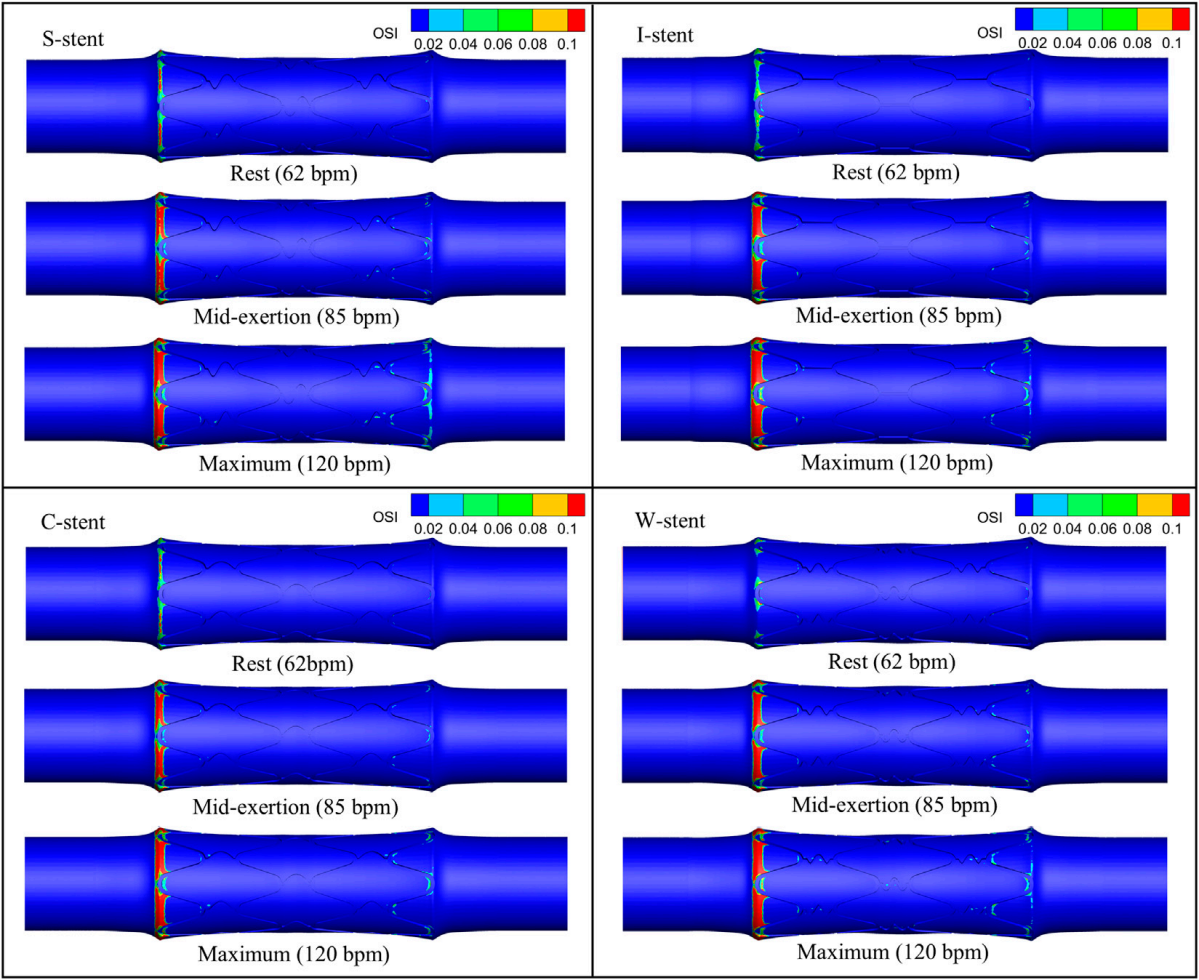
OSI distribution corresponding to all stents under different physiological states.

**FIGURE 10:**
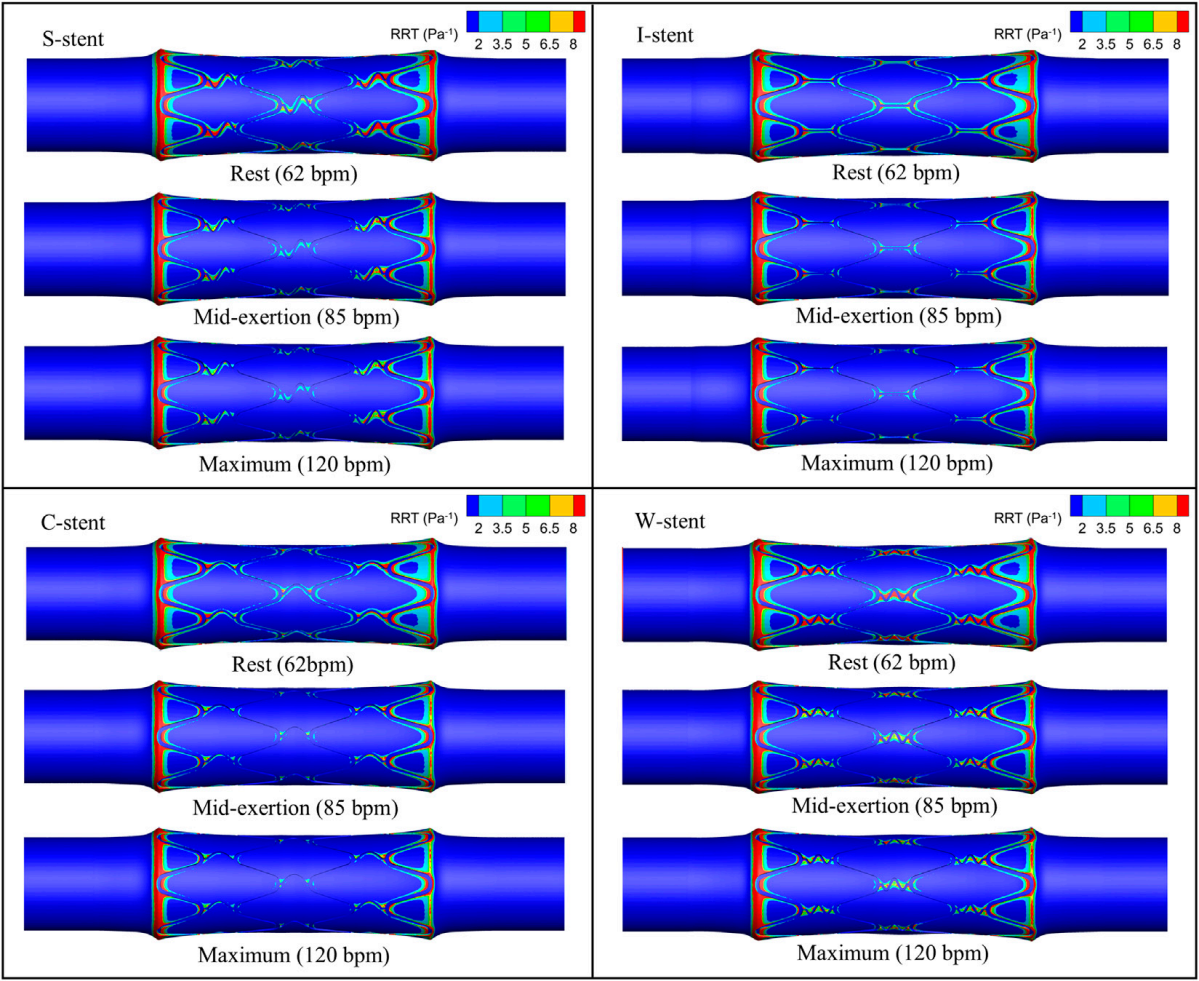
RRT distribution corresponding to all stents under different physiological states.

**FIGURE 11:**
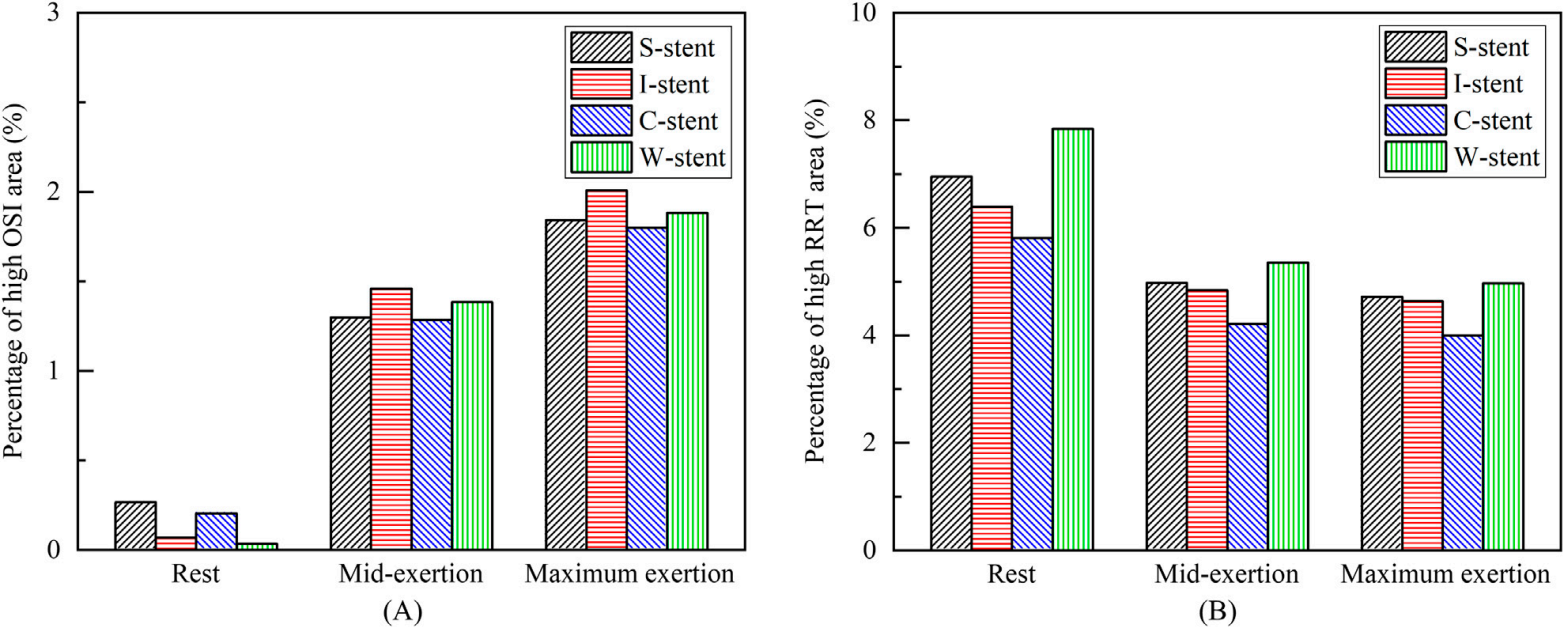
**(A)** Percentage of high OSI areas. **(B)** Percentage of high RRT areas.

The distribution of OSI is shown in Figure 9, where it can be observed that the high OSI region (greater than 0.1) occupies a relatively small area, primarily at the end of the stent near the blood flow inlet. As exercise intensity increases, the high OSI region tends to expand. Furthermore, Figure 10 demonstrates that the high RRT region (greater than 8 Pa^−1^) is mainly concentrated at both ends of the stent and the connector, with the area of the high RRT region decreasing as exercise intensity increases. The area fractions of the high OSI and high RRT regions are shown in Figures 11A, B, respectively, where it can be seen that, with increasing exercise intensity, the OSI area fraction rapidly increases, while the RRT area fraction rapidly decreases. As exercise intensity continues to rise, the rate of change in both OSI and RRT area fractions slows down, which is consistent with the distribution trend of TAWSS shown in Figure 8A. Additionally, while the C-stent exhibits a higher area fraction of OSI at rest, suggesting uneven blood shear stress under resting conditions, it demonstrates more stable hemodynamic characteristics under moderate and intense exercise, with both OSI and RRT area fractions lower than those of other stent designs. These findings indicate that the C-stent may offer superior hemodynamic performance under exercise conditions, potentially reducing the risks of thrombosis and restenosis, and may be particularly beneficial for patients with higher levels of physical activity.

## 5 Discussions

The results demonstrate that the S-stent, I-stent, and W-stent exhibit relatively similar dog-boning and recoiling behaviors; however, the I-stent induces more severe stress concentration in the plaques, which could lead to vascular injury. Overall, the S-stent and W-stent perform better in terms of the structural coupling response. Furthermore, the dog-boning effect does not cause significant stress concentration at the plaque ends, as the ends of the stent only fit the plaque during dog-boning, resulting in minimal compression of the plaque. As the expansion process progresses, the dog-boning effect diminishes significantly, which suggests that a gradual adaptation of the stent to the plaque during the initial expansion may be beneficial in minimizing the adverse effects of dog-boning.

In this study, both low time-averaged wall shear stress (TAWSS) and high oscillatory shear index (OSI) areas are primarily located at or near the ends of the stent, consistent with the findings of Martin et al. (2014) and Wei et al. (2019). Under different physiological conditions, the I-stent and C-stent exhibit superior hemodynamic characteristics compared to the S-stent and W-stent, likely due to the less obstructive connector geometry of the I-stent and C-stent. It is also observed that the low TAWSS region at the connector of the C-stent is predominantly confined to one side, while the low TAWSS region in the I-stent is distributed on both sides of the connector. This difference may explain the relatively lower percentage of low TAWSS areas in the C-stent. In addition, the study demonstrated that mid-exertion (85 bpm) can significantly reduce the low TAWSS and high RRT areas, while further elevation of exercise intensity (maximum exertion, 120 bpm) has a limited effect on reducing the low TAWSS and high RRT areas.

This study has several limitations that need to be considered. First, the geometric models of the vessels and plaques are somewhat idealized, assuming the materials to be incompressible, isotropic, and hyperelastic. While this assumption simplifies the model, it may differ from the heterogeneous and anisotropic characteristics of actual biological tissues, potentially introducing some errors. Additionally, we recognize that the motion of muscles and bones could lead to periodic changes in the shape of blood vessels, which in turn might affect hemodynamic parameters such as TAWSS and blood flow distribution. Particularly during physical activity or other physiological states, the flexibility and dynamic response of the vessel could significantly alter hemodynamic characteristics. However, due to the complexity of modeling these interactions and the lack of detailed physiological data, we have simplified the vessel wall as rigid in our model, without considering its deformation under dynamic conditions. Nevertheless, previous studies (such as Chiastra et al., 2014) have shown that for simulating hemodynamic parameters like TAWSS, the trends obtained with the rigid wall assumption are similar to those from flexible wall models. Therefore, we believe the current model is sufficient to capture the main effects of stent design on blood flow. Future research, however, should extend the existing models to account for dynamic deformation of the vessel walls and the influence of muscle and bone motion on hemodynamics. The introduction of more refined fluid-structure interaction models (Qiao et al., 2022; Qiao et al., 2023), high-resolution imaging techniques (such as dynamic magnetic resonance imaging), and biomedical radar sensor technologies (Qiao et al., 2022) could provide a more comprehensive simulation of coronary behavior under real physiological conditions, further enhancing our understanding of how factors like motion impact vascular function. Such studies will offer more precise guidance for stent design, particularly in personalized treatments and long-term outcome prediction.

## 6 Conclusion

The structural coupling model comprising the balloon, stent, plaque, and artery is developed, with the fluid domain model derived from the deformed structure. The mechanical properties and hemodynamic characteristics of the stent with four connectors are analyzed, and the key findings are summarized as follows.

The study demonstrates that the S-stent, I-stent, C-stent, and W-stent all exhibit a pronounced dog-boning phenomenon when expanded by the balloon. The maximum stress in the stent occurs at the junctions of the rings and connectors at the ends of the stent upon initial contact with the plaque. As the stent expands, the maximum stress gradually shifts from the ends of the stent toward the center. Among the stents, the S-stent and W-stent show relatively low dog-boning (*F*_d_) and recoiling (*F*_r_), resulting in minimal damage to both the plaque and artery during expansion.

The areas of low TAWSS and high RRT are primarily located near the ends of the stent, with the low TAWSS regions showing an overall decreasing trend as the heart rate increases. The C-stent exhibits the smallest proportion of low TAWSS and high RRT areas across different physiological states. Notably, the low TAWSS and high RRT areas significantly decrease when transitioning from rest to moderate exertion, while the rate of reduction is much slower when shifting from moderate exertion to maximum exertion. These findings provide critical insights for the design of stent connectors and offer valuable guidance regarding exercise intensity for patients undergoing stent intervention.

## Data Availability

The original contributions presented in the study are included in the article/supplementary material, further inquiries can be directed to the corresponding author.
